# On Physiognomy

**Published:** 1918-11-09

**Authors:** 


### ON PHYSIOGNOMY.

By CADUCEUS.

ONE of the advantages of the present commercial state of medicine is that it brings a sure living: one of the disadvantages, that it is so partially explored. Anything with a close bearing on therapeutics is done to death, and anything without is cold-shouldered. One of these neglected but philosophical by-paths is physiognomy—not the physiognomy of disease, but physiognomy as a branch of ethology, physiognomy as an index to character. Hitherto this has been largely the affair of the literary author, especially the novelist. How well we know that "strong man" with the big jaw, mostly firmly clenched, sometimes "thrust forward," like Captain Kettle's—how well, too, the "weak mouth"! And the brows, stern, rugged, projecting, always "like a penthouse," beneath which, as from a sheath, the eye flashes like a falcon's. Then there is the Iron Duke's egotistical praise of plenty of nose with a high bridge to it, while we have it from Dumas (as he probably, born copyist that he was, from somebody else) that big cheekbones unfailingly mean craft and audacity, as we saw in the case of my late lamented acquaintance the Humbug. But we never hear *why* strongly-marked features should indicate strength of purpose. Well, to me the explanation seems simple. All the stigmata noted above agree in being advantageous in a physical combat *corps à corps* such as very primitive man engaged in. The massive jaw means a deadly bite—look at the under-jaw of the Piltdown skull, or of that prehistoric cranium lately discovered in Australia. Rugged brows, a high nasal bridge, spreading malar eminences, all serve to protect that vital organ the eye. Modern civilisation has done away with the necessity for actual biting, but it still gives its minor prizes not to capacity, but to force, or, as the expressive synonym goes, to "cheek"; and so people with a primitive combative faculty usually take them. The highest positions were, of course, unattainable through mere scrapping as soon as man became more human than simian, and accordingly the great figures of humanity are not characterised as has been described. Consider the busts in the hall of our own College of Surgeons. The two greatest there, John Hunter and Lister, have Socratic noses, as Darwin had. But when you go lower, where capacity had to be supplemented, if ever so little, with self-assertion, then you get "strength" beginning to show itself in the familiar directions. Sir James Paget, Sir Charles Bell, Sir William MacCormac have the high bridge. Even the great Huxley's snub nose was spoilt by a powerful jaw and rat-trap mouth. Mentioning Huxley brings to mind, however, his saying about the tragedy of a theory slain by a fact. Be it granted at once, then, that great artists, at all events great plastic and pictorial artists, usually have aquiline noses. Hokusai had, and the Japanese, as a nation, have not much feature. Also they are large-browed, the famous "bar of Michael Angelo," which looks to be just big frontal sinuses. There are two ways of accounting for this phenomenon, of which the first rather goes to prove the rule laid down to begin with. For Freud—I have not seen Freud's likeness—says that the mind of the artist is like the mind of the savage. (If you doubt this, engage in the enviable task of reading the works of William Butler Yeats, far the finest poet in English of this generation.) The second hypothesis is that a great sculptor or painter or architect must needs possess some qualities of the man of action, for he has not to meditate only, but to carry out physical operations. And it almost seems as if, among artists too, pure facial type is only to be found amongst the first-raters. Just as the lesser medical stars show "strong" features, so do lesser artistic ones "weak" features. There is a collection of portrait photographs of English literary and artistic celebrities done by Mr. A. L. Coburn, and perhaps of considerable merit; certainly it makes a good drawing-room book. Of course amongst his sitters there is not likely to be more than one man of genius. Well, what strikes you is the ordinariness of appearance; here a painter of fame with the fat face of a grocer, there another with the self-satisfied look on his mean little features of a clerk in the Margate train showing round photographs of his backyard. Do not, of course, take me to mean that mere distinction of countenance is the mark of artistic pre-eminence. Sir Johnston Forbes Robertson has that, and his art, acting, is the lowest of all.

But here, perhaps, had better end this plunge of a mere doctor into the deep waters of humanism.

